# Prevalence and correlates of depressive symptoms among professional drivers in Saudi Arabia: a cross-sectional study

**DOI:** 10.1192/bjo.2021.751

**Published:** 2021-06-18

**Authors:** Adnan Raed Alnaser, Osama A. Zitoun, Tawfik Rajab, Abdullah Khojah, Juliann Saquib, Nazmus Saquib

**Affiliations:** College of Medicine, Sulaiman Al Rajhi University

## Abstract

**Aims:**

Due to the nature of their work, professional drivers face a considerable risk of developing depression and other mental illnesses. We sought to assess the prevalence and the factors influencing depressive symptoms among professional drivers in Saudi Arabia.

**Method:**

Using convenience sampling, we have conducted an interviewer-administered survey on 324 professional drivers in Qassim Region in Saudi Arabia using Depression subscale from the Depression, Anxiety and Stress Scale 21 (DASS-21). Participants were interviewed in their native language, and responses were outlined directly into an online form in English. Data were then extracted and analyzed using SPSS software.

**Result:**

Participants’ mean age was 38.6 years, and mean driving hours per day were 9.86 hours/day. The mean DASS-21 depression score among the professional drivers was 2.88. Overall, 21.9% of the included drivers had variable degrees of depressive symptoms, with 7.4% suffered from extremely severe symptoms. Depressive symptoms were influenced by the driver's nationality, educational level, vehicle type, driving years, BMI, presence of chronic medical conditions, physical activity, and sexual activity. Moreover, poor sleep quality increased the risk of developing depressive symptoms among the drivers by 31.9 times (OR: 31.9, CI: 9.03–112.63, P < 0.001).

**Conclusion:**

Nearly one-fifth of professional drivers in Saudi Arabia (Qassim region) suffer from depressive symptoms. Unhealthy lifestyle practices (i.e. being obese and physically inactive) have been closely related to depressive symptoms. Education, sexual activity, type of driven vehicle, and the number of chronic conditions were also associated with depressive symptoms. Also, poor and fair sleep quality was strongly associated with the development of depressive symptoms as compared with excellent sleep quality. As drivers are always on the move and hardly reachable, we would propose psychological support and counseling to be administered via telemedicine services. Future research is needed to better comprehend the needs of this vulnerable population.

